# L’observance thérapeutique: un autre défi à relever chez l’hémodialysé chronique

**DOI:** 10.11604/pamj.2019.33.28.9448

**Published:** 2019-05-15

**Authors:** Saad Alaoui Mhammedi, Fathia Hamdi, Mohammed Benabdelhak, Yassamine Bentata, Intissar Haddiya

**Affiliations:** 1 Centre Hospitalier Universitaire Mohammed VI, Oujda, Maroc

**Keywords:** Observance thérapeutique, hémodialysé chronique, insuffisance rénale chronique, Pregnancy not followed, antenatal care, maternal and perinatal outcomes, Lubumbashi

## Abstract

La mauvaise observance thérapeutique (OT) est fréquemment rencontrée dans la plupart des maladies chroniques. Elle accroît la morbi-mortalité et les dépenses de santé. Les patients hémodialysés chroniques ont souvent une OT peu satisfaisante. L'objectif de notre étude était d'évaluer le niveau d'OT chez nos patients hémodialysés chroniques et d'identifier les facteurs prédictifs de mauvaise compliance. Nous avons réalisé une étude transversale en novembre 2011 au centre hospitalier d'Oujda. L'OT fût mesurée à l'aide d'un questionnaire: test d'évaluation de l'observance (TEO) tenant compte à la fois de l'adhésion au traitement médicamenteux et au régime alimentaire. Un taux de 85% était retenu arbitrairement comme seuil de bonne OT. Un taux entre 57% et 85% permet de situer le patient dans une zone d'observance partielle, et un taux inférieur à 57% indique la mauvaise OT. Les différents paramètres démographiques et clinico-biologiques ont été analysés et nous avons dégagé les facteurs prédictifs de bonne et mauvaise OT. Notre étude a porté sur 101 patients hémodialysés chroniques, le sexe ratio est de 1,2. La moyenne d'âge 49,7 ± 15,6. Le TEO a objectivé 23,4% de bons observants, 39,4% d'observance partielle et 37,2% de mauvais observants. L'analyse statistique a montré que la mauvaise OT est associée au bas niveau socio-économique et intellectuel et la présence de comorbidités et la longue durée d'hémodialyse. La bonne OT a été observée chez les patients d'âges extrêmes aidés par une tierce personne, le nombre réduit de médicaments et le niveau intellectuel.

## Abstract (en)

Poor compliance with therapy is frequently encountered in most of patients with chronic diseases. It increases the risk of morbi-mortality and healthcare costs. Patients on chronic haemodialysis often have poor compliance with treatment. This study aims to assess the level of compliance with treatment in our patients on chronic hemodialysis and to identify the predictive factors of poor compliance with treatment. We conducted a cross-sectional study at the Oujda Hospital in November 2011. The compliance with treatment was measured using a questionnaire: the compliance evaluation test (CET) assessed the compliance with medication treatment and diet. A rate of 85% was retained arbitrarily as a threshold for good compliance with treatment. Patient in a zone of partial adherence had a rate between 57% and 85% while a lower rate (57%) indicated poor compliance with treatment. Different demographic and clinicobiologic parameters were analyzed and predictive factors for good and poor compliance with treatment were identified. Our study involved 101 patients on chronic haemodialysis; the sex ratio was 1.2, the average age of patients was 15.6 years. CET showed that 23.4% of patients had good compliance with treatment, 39.4% partial compliance with treatment and 37.2% poor compliance with treatment. Statistical analysis showed that poor compliance with treatment was associated with a lower socio-economic and intellectual status, with co-morbidities and with long term hemodialysis. Good compliance with treatment was observed in very old patients helped by a third person, taking a reduced number of medications, with a higher intellectual level.

## Brève

**Introduction**: la mauvaise observance thérapeutique (OT) se rencontre dans la plupart des maladies chroniques; ou il se crée souvent un lien intime et ritualisé entre le médicament et son« consommateur» chronique [1]. En effet, la décision du patient d'adhérer ou non au traitement prescrit peut être inconsciente ou intentionnelle [2]. Le mauvaise compliance involontaire est souvent due aux limites et contraintes propres au patient à type d'oubli, manque d'information et/ou d'assimilation. À l'inverse, La mauvaise observance intentionnelle exprime généralement un rejet voire un refus des recommandations des soignants. Les patients hémodialysés chroniques (HDC) ont souvent une OT peu satisfaisante. Ce qui aggrave d'autant plus la morbi-mortalité chez ces patients.

**Figure 1:**
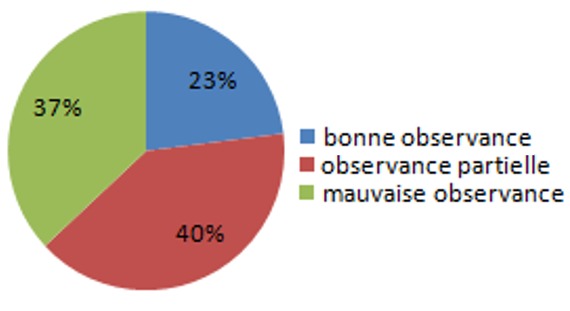
Taux de l’observance thérapeutique

**Méthodes**: c'est une étude transversale, réalisée en novembre 2011 au centre d'hémodialyse du centre hospitalier d'Oujda, incluant 94 patients hémodialysés. L'OT fût mesurée à l'aide d'un questionnaire: test d'évaluation de l'observance (TEO) tenant compte de l'adhésion au traitement médicamenteux et au régime alimentaire; le questionnaire auquel répondaient les patients contenait à la fois des questions générales relatives aux données socio-démographiques et aux données médicales pertinentes au regard de notre étude. Nous avons conçu un questionnaire basé en grande partie sur le test d'évaluation thérapeutique de Girerd. En absence d'une version marocaine du test d'évaluation thérapeutique de Girerd au début de l'étude, le questionnaire (origine) a été traduit et adapté au contexte Marocain. Structure du questionnaire: (Annex 1, Figure 1) [3].

**Résultats**: notre étude a inclus 101 patients, l'âge moyen de nos patients était de 49, 7±15,6 (8-82). Une légère prédominance masculine a été (H/F=1,2). La majorité de nos patients avaient un faible niveau d'instruction: 47,26% étaient analphabètes, 34,20% avaient un niveau d'instruction primaire et 4,2% de patients étaient universitaires. Le niveau socio-économique de la majorité de nos patients était assez bas. L'ancienneté moyenne en hémodialyse chez nos patients était de 10, 48±6,23 ans. Les néphropathies étaient de cause chez 54%, des patients, 20% de patients avaient une néphropathie vasculaire, et 6% une néphropathie diabétique (Tableau 1). Le TEO a révélé 23,4% de bons observants (BO), 39,4% ayant une observance partielle et 37,2% de mauvais observants (MO) (Figure 1). La bonne observance thérapeutique est directement proportionnelle à l'âge des patients: BO 54,3±12,2 MO36, 6±23,1 (Les patients âgés aidés par une tierce personne), et elle est inversement proportionelle à l'ancienneté de l'HDC BO56, 2±34,2 MO123,1 1±21,5 et la comorbidité cardio vasculaire BO 14% MO 45%. La complexité et le nombre des médicaments sont aussi des facteurs déterminants de l'observance thérapeutique: plus le nombre de médicaments par jour augmente plus la qualité de l'observance diminue BO: 2,1±2,4 MO 4, 09±0,13. Le niveau intellectuel élevé est facteur prédictif de la bonne observance thérapeutique (Tableau 2).

**Tableau 1:** Caractéristiques socio démographiques chez nos patients

Nombre de patients (n)	101
Age moyen (années)	49,7±15,6 [8-82]
Sexe ratio (H/F)	1,2
Niveau d’instruction	
Universitaire	4,2%
Secondaire	8,5%
Primaire	34,04%
Analphabète	47,26%
Néphropathie initiale: indéterminée	54%
Diabétique	6,4%
Néphropathie vasculaire	19,35%
Glomérulonéphrite chronique	20%
Durée moyenne d’HD (années)	10,48±6,23

**Tableau 2:** analyse statistique du taux de l’observance thérapeutique

Paramètres	Bonne OT (23,4%)	OT partielle (39%)	Mauvaise OT (37,2%)	P
Age (années)	54,3± 12,2	42,1±14,3	36,6±23,1	0,02
Niveau d’instruction bas	24,2%	22,6%	53,2%	0,001
Nombre moyen de médicaments	2,1±2,4	3,2±1,5	4,09±0,13	0,0001
Ancienneté en HDC (mois)	56,2±34,2	97,1±21,5	123,1 1±21,5	0,003
Comorbidités cardio-vasculaires (%)	14%	32,12%	45%	0,01

**OT:** Observance thérapeutique; **HDC:** hémodialyse chronique

**Discussion**: nos résultats ont montré que les facteurs de risque d'une mauvaise observance thérapeutique sont l'âge jeune des patients, le niveau d'instruction bas, le nombre moyen de médicaments l'ancienneté en hémodialyse et présence de comorbidité. Une enquête menée en Afrique sub-Saharienne, analysant le taux de l'observance thérapeutique chez les patients atteints de pathologies rénales chroniques a retrouvé comme facteurs de mauvaise observance: l'oubli, défaut de l'information médicale, complexité de la posologie, l'inaccessibilité du système de soins, prise de phytothérapie, les effets indésirables des médicaments et leur cout élevé [4]. Chez les sujets âgés, les modifications physiques et cognitives auxquels se rajoute la fréquence élevée des effets secondaires et la polymédication, sont des freins à la bonne observance [5]. Cependant, dans notre étude l'âge était un facteur de bonne observance vu que la majorité dans sujets âgés, dans notre société, sont aidés par une tiers personne. Des études récentes incriminent d'autres facteurs épidémiologiques dans la mauvaise observance thérapeutique à titre d'exemple: la race noire reste la moins adhérente aux traitements [6].

Une bonne observance est observée chez les hommes à contrario des femmes [7]. L'état psychique du patient constitue un facteur déterminant de l'observance au traitement. La dépression, les troubles de comportement et les l'anxiété sont des handicaps pour la bonne observance [8]. Différents études ont montrées la présence d'une relation intime entre le bas niveau d'instruction et la mauvaise observance thérapeutique, résultat qu'on a pu également démontrer dans notre série. En effet, les patients ayant un niveau culturel bas, sont dans l'incapacité de comprendre l'importance des traitements surtout que ses bénéfices pour les maladies chroniques sont des bénéfices à long terme [9]. Le taux de l'observance thérapeutique reste inversement proportionnel au nombre de prises médicamenteuses par jour dans les différents séries. Donc, le prescripteur doit tenir compte de la nécessité de la réduction du nombre des prises journalières. Le cout financier constitue également une barrière à l'adhésion au traitement aussi bien dans les pays développés que dans les pays en voie de développement [10]. Chez les patients sans sécurité sociale, plusieurs problèmes leur semblent prioritaires que la médication (logement, alimentation…). Kauz *et al*. ont montré que les insuffisants rénaux avec un niveau socio-économique bas ont moins accès aux structures de soins et commencent tardivement la dialyse. Les effets indésirables du traitement sont également une barrière supplémentaire à la mauvaise observance thérapeutique [4]. La non observance est également déterminée par la pathologie. L'observance diminue progressivement avec la durée d'évolution de la maladie ce qui pose un problème majeur dans les prises en charges des pathologies chronique. En effet, il a été démontré que chez les transplantés rénaux, l'observance des traitements immunosuppresseurs décroit à neuf mois pour atteindre 48% à un an [2]. Le patient n'est pas le seul en cause. L'observance est une responsabilité collective [11].

**Conclusion**: l'observance thérapeutique chez les sujets atteints de pathologies rénales chroniques pose un défi. La majorité des facteurs de mauvaise observance recensés peuvent être évités par une bonne coordination entre le patient, son médecin traitant, le personnel soignant et l'entourage. La meilleure connaissance des facteurs de la non observance permettra de les éviter.

## Conflits des intérêts

Les auteurs ne déclarent aucun conflit d'intérêts.

## Annexes 1 Test d’évaluation de l’observance

- Ce matin, avez-vous oublié de prendre votre médicament ?
  - Depuis la dernière visite, avez-vous été en panne de médicament?
  - Depuis la dernière visite, avez-vous consommé des aliments déconseillés par vos soignants?
- Vous est-il arrivé de prendre votre traitement avec retard par rapport aux horaires habituels?
- Vous est-il arrivé de ne pas prendre votre traitement parce que certains jours votre mémoire vous fait défaut?
- Vous est-il arrivé de ne pas prendre votre traitement parce que certains jours vous avez l’impression que votre traitement vous fait plus de mal que de bien?
  - Pensez-vous que vous avez trop de comprimés à prendre ? Pensez-vous que votre régime alimentaire est astreignant?
